# Effective Community Engagement during the Environmental Assessment of a Mining Project in the Canadian Arctic

**DOI:** 10.1007/s00267-021-01426-5

**Published:** 2021-02-02

**Authors:** Jason Prno, Matthew Pickard, John Kaiyogana

**Affiliations:** 1 Jason Prno Consulting Services Ltd., Peterborough, ON K9H 3R5 Canada; 2 Sabina Gold & Silver Corp., Vancouver, BC V7X 1M9 Canada; 3 Sabina Gold & Silver Corp., Cambridge Bay, NT X0B 0C0 Canada

**Keywords:** Community engagement, Corporate social responsibility, Environmental assessment, Mining, Arctic, Canada

## Abstract

The Back River Project is an approved gold mine in Nunavut, Canada owned by Sabina Gold & Silver Corp. Sabina developed a comprehensive community engagement program during the environmental assessment phase of the Project to share information, receive and address local feedback and concerns, and develop productive relationships in support of Project advancement. This paper outlines Sabina’s engagement program, successes and challenges encountered from the perspective of a mineral developer, and insights obtained for effective community engagement in a Canadian Arctic context. The program has been commended by observers and is consistent with best practice models. Sabina’s experiences revealed the importance of engaging early and often using a context-specific approach; comprehensive record-keeping and reporting; the meaningful incorporation of community perspectives and Traditional Knowledge; and focusing on long-term relationships, partnerships, and local benefits. Effective community engagement subsequently played a key role in Sabina securing major licenses and permits for Project advancement.

## Introduction

The Back River Project (Project) is an approved gold mine in Nunavut, Canada owned by the Canadian company Sabina Gold & Silver Corp. (Sabina). The Project is comprised of two main areas with an interconnecting Winter Ice Road: the Goose Property where open pit and underground mining operations will occur in addition to ore processing, and a Port and Marine Laydown situated ~130 km to the north. Prior to commencing construction and operations, Sabina was required to complete a multi-year environmental assessment (EA) process overseen by the Nunavut Impact Review Board (NIRB). EA is one of the most influential aspects of environmental regulation and policy in North America. It involves describing a proposed activity and applicable baseline conditions, possible environmental and socio-economic effects of the activity, measures to mitigate or eliminate adverse effects while providing benefits, identification of remaining impacts and their significance, and plans for follow-up and monitoring. Regulatory authorities then decide whether to accept, reject, or modify the project proposal (Hanna 2016).

**Fig. 1:**
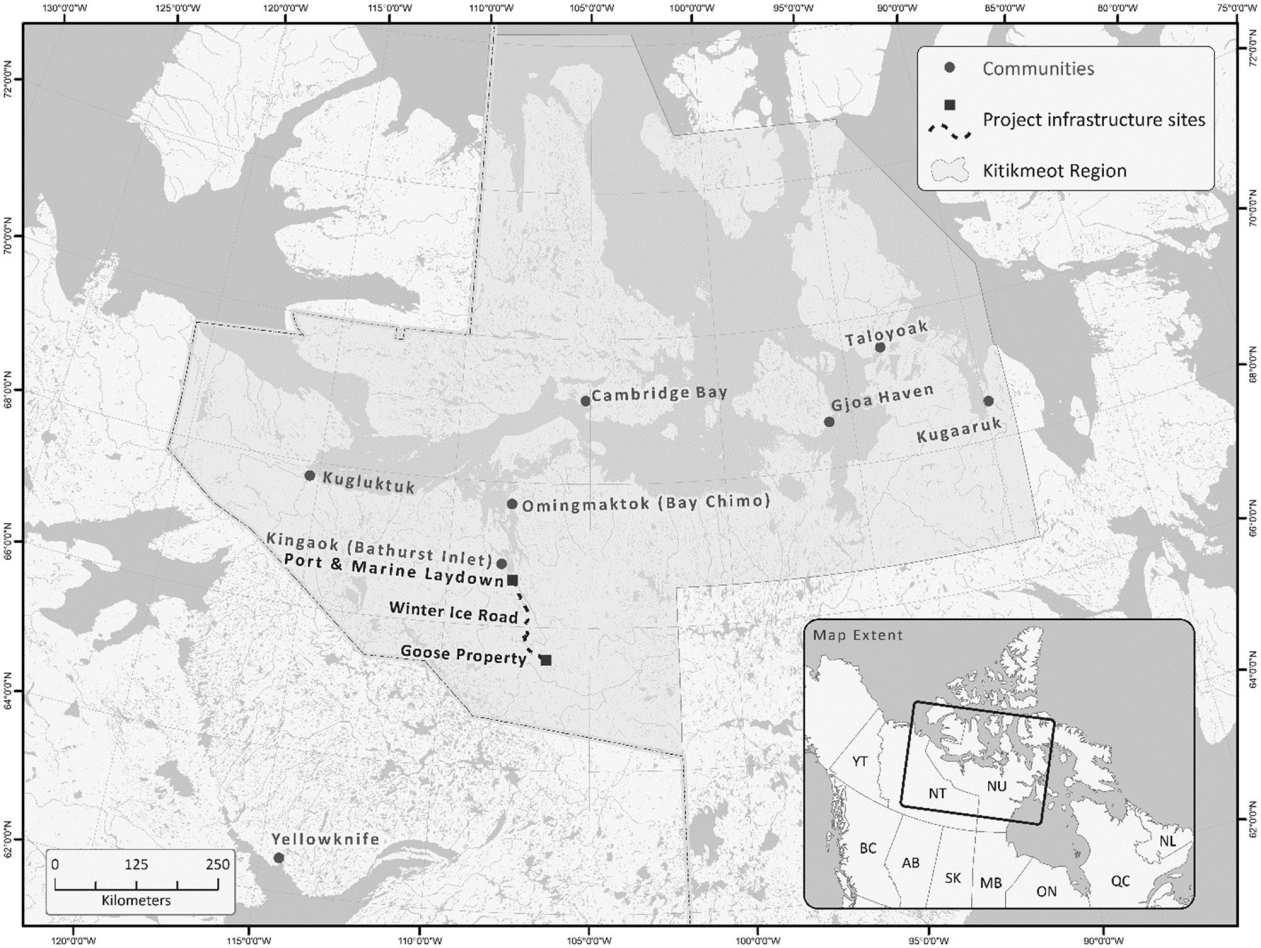
Location of the Back River Project and surrounding communities

As part of the Project’s EA process, Sabina developed a comprehensive community engagement program to share information, receive and address local feedback and concerns, and develop productive relationships in support of Project advancement. Due to their traditional and socio-economic ties to the Project area, Sabina focused its engagement efforts on the Kitikmeot Region communities of Cambridge Bay, Kugluktuk, Bathurst Inlet, Bay Chimo, Gjoa Haven, Taloyoak, and Kugaaruk (Fig. 1).^1^ Approximately 90% of the Kitikmeot Region’s population of 6900 are Inuit (NBS 2019; Statistics Canada 2017). Inuit are Indigenous peoples of Canada’s Arctic and one of three Indigenous groups officially recognized in Canada’s Constitution Act, 1982 (the others being First Nations and Métis). Inuit culture and traditions remain vibrant in Nunavut. Strong connections to the natural environment also exist and subsistence harvesting continues to be practiced regularly. However, Inuit communities face various socio-economic challenges compared to the rest of Canada, including high unemployment and social assistance rates, low levels of educational attainment, and deficits in several other health and well-being indicators (ITK 2014).

Sabina understood the value effective community engagement would bring to its EA and was familiar with the various legal requirements and stakeholder expectations surrounding it. The importance of community engagement in the EA process is recognized both internationally and throughout the Canadian Arctic (André et al. 2006; Noble 2015; Sinclair and Doelle 2015, Udofia et al. 2015; Vanclay et al. 2015; Hanna 2016; Arctic Council 2019). Effective engagement ensures community members are informed about proposed projects and their concerns are more readily addressed. It thus contributes to the improved substance and acceptability of decision making. The process of decision making is also improved when the public is involved (Funtowicz and Ravetz 1993; Eden 1996; Barton 2002; Pring and Noe 2002; Innes and Booher 2004; Parkins and Mitchell 2005; O’Faircheallaigh 2009; Diduck et al. 2015; Mitchell 2019). Although government and regulatory agencies often have consultation mandates to fulfill, proponent-led engagement programs are an important mechanism through which communication and decision making often occurs in practice. Effective engagement by proponents is also crucial during EA, as this is when regulators will carefully evaluate the degree to which local concerns have been addressed before issuing major authorizations.

Formal requirements for community involvement in resource development exist in Nunavut, with the Nunavut agreement (NA) establishing notable precedents. The NA is a comprehensive land claims agreement signed in 1993, whereby Inuit exchanged title to their traditional land in the Nunavut Settlement Area for a series of rights and benefits.^2^ Designated Inuit organizations, such as the Kitikmeot Inuit Association (KIA), then gained important authority related to resource development in their regions, including land ownership and management, issuance of permits and other authorizations, and the ability to negotiate Inuit Impact and Benefit Agreements (IIBAs) with proponents.

The NA also created the NIRB, which is a resource co-management institution of public government whose authority is defined in the Nunavut Planning and Project Assessment Act (NuPPAA). NIRB is the sole agency overseeing EA in Nunavut and was created to ensure Inuit have an opportunity to be formally involved in, and even direct, impact assessment in Nunavut (Barry et al. 2016; NIRB 2020).^3^ The nature of the land claims agreement on which the Nunavut EA process rests means that Indigenous communities play an even stronger role in the process than they would in Canadian federal and provincial EAs (Sinclair and Doelle 2015). Evolving legal obligations related to Indigenous consultation have also created additional pressures on industry (Prno and Slocombe 2012; Mulrennan 2015).

Furthermore, the concept of a Social License to Operate has emerged to describe what stakeholders themselves have come to expect from mining projects and outlines the broad parameters of what proponent-led community engagement should aim to accomplish. A social license exists when a mining project is seen as having the broad, ongoing approval and acceptance of society to conduct its activities (Joyce and Thomson 2000; Thomson and Boutilier 2011). Gunningham et al. (2004: 307) add that it “governs the extent to which a corporation is constrained to meet societal expectations and avoid activities that societies (or influential elements within them) deem unacceptable, whether or not those expectations are embodied in law.” The granting of a social license often implies local communities have been meaningfully involved in decision making, have had their concerns substantively addressed, and have received sufficient benefit from a mining project. When community support for resource developments has lacked, project advancement and regulatory approval challenges have frequently occurred in northern Canada (e.g., Poelzer 2002; Bone 2009; White 2009; Prno and Slocombe 2012; Mulrennan 2015; Brown 2019). Protests, blockades, legal challenges, and interventions in regulatory reviews are some of the tools unsupportive communities have used.

Sabina sought to develop a comprehensive community engagement program to address this complex social and regulatory landscape. For the purposes of this paper, three criteria were useful for assessing the “effectiveness” of this program over time: (1) whether broad community support for the Project (i.e., a social license) was obtained; (2) whether an EA approval was issued; and (3) whether tangible links between a social license and EA approval could be discerned. The remainder of this paper describes Sabina’s engagement program, successes and challenges encountered from the perspective of a mineral developer, and insights obtained for effective community engagement in a Canadian Arctic EA.

### Sabina’s Community Engagement Program

Sabina’s community engagement program was multi-faceted. It included the use of various engagement strategies and tools identified in the best practice literature (e.g., ITK and NRI 2007; Government of Nunavut 2012; PDAC 2013; Li et al. 2014; Diduck et al. 2015; ICMM 2015; NIRB 2020), and a commitment to cultural sensitivity and inclusiveness. Bowen et al. (2010) developed a typology of three primary engagement strategies available to companies: transactional, transitional, and transformational engagement. These exist on a continuum, with transactional forms having the least amount of community engagement and transformational forms having the most. Each of these strategies has different applications and merits, and Sabina employed several tools within each strategy to ensure a wide range of individuals were engaged through diverse means. This also enabled key stakeholder views regarding the Project to be better understood and addressed (Table 1).

**Table 1:** Types of community engagement employed by Sabina (adapted from Bowen et al. 2010)

Type of community engagement strategy	Description	Back River Project examples
Transactional	Information exchange and community investment.	• Project newsletters
		• Community posters
		• Social media (e.g., website, email, Twitter)
		• Other distribution materials
		• Information booths (e.g., trade shows)
		• Community donations
		• Sabina’s Cambridge Bay office
		• Community advertisements
Transitional	Two-way communication, consultation, and collaboration, but largely controlled by the company.	• Public meetings
		• Meetings with key stakeholders/groups (e.g., hamlets, hunters and trappers organizations)
		• Community advisory groups (CAGs)
		• Community Liaison Officer (CLO)
		• Mine site visits
		• Radio shows
		• Local employees and contractors
		• Socio-economic and land use studies
		• Tradeshow participation and presentations
		• Cross-cultural training
Transformational (“partnerships”)	Joint project management and decision-making, two-way communication and dialogue, frequent interaction, and the sharing of benefits and outcomes with local communities.	• Inuit Impact and Benefit Agreement (IIBA)
		• Regional and Project-specific Traditional Knowledge studies
		• Bernard Harbour Restoration Project

Successful use of these tools played a key role in Sabina securing major licenses and permits necessary for Project advancement. Most importantly, after a comprehensive five-and-a-half year EA process, Sabina received its Project Certificate from NIRB in December 2017. Leading up to this milestone, some 250 community and stakeholder meetings were held on the Project in addition to numerous other activities. Sabina then finalized an IIBA and long-term land tenure agreements with the KIA in April 2018. The Project has received broad community support in Nunavut and its engagement program has been commended by several observers, including NIRB (2017a: 25–27) in its Revised Final Hearing Report:

As recognized by the Intervenors and Community Representatives who participated in both the 2016 Final Hearing and the supplemental Final Hearing, Sabina’s FEIS Addendum and revisions to the Proponent’s plans are substantive and were the product of extensive consultation with Intervenors and communities… Further, the Board commends the collaborative approach Sabina and all participants took… there was wide-spread support for the Project from all Intervenors, Community Representatives and most members of the public in attendance… the board is now optimistic the Project will represent a model of sustainable development that protects the ecosystemic integrity of the region, integrates Inuit Qaujimaningit and Traditional Knowledge into the Project on an on-going basis, and delivers significant and sustained socioeconomic benefits to Nunavummiut…

The Project was also highlighted in the Arctic Council (2019) document “Good Practices for Environmental Impact Assessment and Meaningful Engagement in the Arctic”. This case study documents the substantial stakeholder support Sabina developed during the EA and the important influence community perspectives had on the design of the Project. However, it also describes NIRB’s initial recommendation for the Project not to proceed and highlights opposition certain stakeholders in the Northwest Territories had. While this June 2016 NIRB recommendation “came as a surprise to many” (Arctic Council 2019: 36), including Sabina, and the Government of Canada (2017a: 3) had noted “many of the participants (including Indigenous and non-Indigenous witnesses and subject matter experts) expressed confidence that the measures presented could mitigate and manage potential adverse effects to an acceptable level”, Project approval was nevertheless left in limbo.

NIRB made its original recommendation “on the basis of the potential for significant adverse ecosystemic and socio-economic effects in Nunavut and also in the Northwest Territories that, in the Board’s view, cannot be adequately managed and mitigated…”. NIRB also concluded, “effects on caribou and terrestrial wildlife could result in additional cumulative and transboundary effects on already declining populations” (NIRB 2016a: 3). Caribou were a key issue considered throughout the review, as it is a culturally valued keystone species and primary subsistence resource for local Indigenous harvesters. Substantial declines in regional caribou populations (unrelated to the Project) were also ongoing at the time of the EA and led to increased public scrutiny of potential effects.

Immediately following the initial negative recommendation, Sabina worked with stakeholders to address NIRB’s (2016a) outstanding concerns. Sabina also asked community representatives if they would consider preparing letters of support for the Project to help overturn the recommendation (as final acceptance/rejection of NIRB recommendations is made by a minister(s) of the federal government). In an exceptional response, letters supporting Project advancement were received from the Hamlets of Cambridge Bay, Kugluktuk, Gjoa Haven, Taloyoak, and Kugaaruk; the Kugluktuk Hunters and Trappers Organization (HTO); individuals from Sabina’s Cambridge Bay and Kugluktuk community advisory groups (CAGs); and several members of the public and northern businesses.^4^

The Government of Canada (2017a) subsequently returned the assessment to NIRB for reconsideration, citing deficiencies in NIRB’s (2016a) Final Hearing Report and premature conclusions that it reached. As part of the reconsideration process that followed, further engagement activities were held by Sabina, impact mitigation plans were refined, and additional EA materials were submitted. Following the completion of a Supplemental Final Hearing, NIRB reversed its original decision and recommended the Project proceed in July 2017. In support of this, NIRB (2017a: 14) cited “the development of some of the ‘best in class’ caribou protection measures that Nunavut has ever seen” and recognized “the collective efforts of Sabina, the KIA, Elders, harvesters and community members to ensuring that Inuit Qaujimaningit and Traditional Knowledge contributions have been incorporated into this assessment in a meaningful way.”

There is much to be learned from Sabina’s community engagement program, with insights that may benefit other northern mineral developers participating in the EA process. More specifically, Sabina’s experiences revealed the importance of engaging early and often using a context-specific approach; comprehensive record-keeping and reporting; the meaningful incorporation of community perspectives and TK; and focusing on long-term relationships, partnerships, and local benefits. Although Sabina’s engagement program was specific to one particular Canadian Arctic context, insights applicable to a broader audience are also offered. To help substantiate its findings, this paper has relied on the experiences of Project team members, public EA records, the community engagement literature, and other relevant sources.

### Insights for Effective Community Engagement

#### Engage early and often using a context-specific approach

Recent observations clearly demonstrate effective engagement programs are a long-term commitment in northern Canada. Considerable time is often needed for proponents to identify major stakeholders, develop their trust and an understanding of key issues, and address relevant community concerns. Communities themselves typically also require long periods to become familiar with a project and its proponent, consider potential implications on their livelihoods and develop informed views on development proposals. Regulatory timelines for these projects are likewise long, owing (at least in part) to the complexity of these reviews and the many opportunities provided for stakeholder input.

There are no “one size fits all” approaches to community engagement and proponents must tailor their programs to the stakeholders directly impacted by their operations (Li et al. 2014). Each development context is also unique, with social license considerations influenced by a suite of complex factors at local, regional, and other scales (Prno and Slocombe 2014). Sabina addressed important contextual factors throughout its engagement program and initiated discussions with communities early in the EA process.^5^ This allowed appropriate opportunities for dialogue to occur and time for relevant management responses and Project changes to be made during the EA.

Sabina utilized stakeholder theory (Mitchell et al. 1997; Freeman et al. 2007) to prioritize certain communities and stakeholder groups for engagement based on their ability to impact, or be impacted by, Project operations. Initial plans and priorities for community engagement were developed after identifying relevant community ties to the Project area, scoping potential impacts on Project stakeholders, and consulting applicable guidance documents (e.g., past EA records and best practice literature). This assessment resulted in western Kitikmeot Region communities being prioritized for enhanced engagement over others. Engagement plans were then refined over the course of the EA as knowledge was gained, stakeholder feedback was obtained, and potential issues became clearer. For example, increased focus was placed on the seasonal communities of Bathurst Inlet and Bay Chimo once their land use and harvesting ties to the Project area became better understood through detailed TK studies and in-person engagement. Likewise, increased consideration of perspectives voiced by Northwest Territories Indigenous groups occurred once the potential for transboundary effects (e.g., on caribou) became apparent.

Recognizing Inuit communities are diverse and no one engagement method could reasonably reach all affected parties, multiple tools were employed to engage a broad spectrum of community members. This consisted of regular in-person meetings with the general public and key stakeholder groups, community research initiatives, and various audio, visual, and written media methods (see Tables 1 and 2). Opportunities for community members to provide feedback on Sabina’s engagement program were also provided and suggestions were addressed where appropriate.

**Table 2:** Community engagement record for the project’s EA phase

Community	Completed meetings	Attempted meetings	Other engagements
Cambridge Bay	64	6	9
Kugluktuk	61	2	11
Bathurst Inlet/Bay Chimo	11	6	7
Gjoa Haven	19	8	5
Taloyoak	23	1	4
Kugaaruk	18	7	4
Yellowknife/other Northwest Territories location	16	2	25
Regional or other geographic focus	38	1	13
**Total**	**250**	**33**	**78**

“Completed meetings” refers to in-person meetings/events that were planned and successfully completed. “Attempted meetings” refers to in-person meetings/events that were planned or proposed but were unable to be completed. “Other engagements” refers to other major engagement activities that did not include in-person components (e.g., community newsletters, written correspondence, and Project updates)

Sabina’s Sustainable Development Policy further committed the company to promoting a culture of open and meaningful dialogue with stakeholders. In a community engagement context, this entailed being transparent about Project plans and forthcoming with relevant information, acknowledging challenges that were being faced, and welcoming criticism and suggestions offered by others. This approach was the foundation on which Sabina’s relationships with communities were established and was fundamental in developing a successful community engagement program.

Sabina also developed a context-specific approach to community engagement, which took into consideration various factors specific to the Kitikmeot Region and Nunavut more generally. This level of detail was critical for ensuring Sabina’s program was both relevant to local communities and effective. For example, the program took the region’s unique Inuit population, cultural heritage, and geography into account. As Elders and harvesters play valuable and respected roles in Inuit culture, Sabina specifically targeted these individuals through dedicated TK studies (whereby Inuit led the data collection process and owned its outputs, described further below), land use research, regular meetings with local HTOs, and CAGs, site visits, and through general public engagement (e.g., public meetings, radio shows, and newsletters). Likewise, creating employment and training opportunities for youth is a noted priority in Nunavut and Sabina engaged this demographic through meetings with high school students, social media, youth participation on CAGs, and a donations program focused on initiatives pertaining to youth and education. The Cambridge Bay and Kugluktuk CAGs allowed Sabina to engage individuals from key community organizations/demographics (e.g., hamlets, HTOs, Elders, and youth) on a regular basis and were formalized through terms of reference agreed upon by all members.

Working in a cross-cultural setting like Nunavut also presented several challenges, as it created an increased potential for misunderstanding and conflict. Cultural sensitivity measures were employed to help reduce the likelihood of this occurring, including the use of cultural awareness training for company representatives, hiring of a local Community Liaison Officer (CLO), and establishment of a company satellite office in Cambridge Bay. The CLO, who was Inuit, reported directly to a Sabina executive and was responsible for executing many day-to-day consultation and engagement tasks. They were an invaluable resource on Inuit culture and the daily lives of Kitikmeot communities, a key Sabina contact person for local community members, and provided feedback to Sabina managers about issues raised at the community level. The broader community engagement team also possessed many years of experience working with Indigenous communities in northern Canada and had substantial knowledge of Inuit culture and customs.

Sabina’s program also took Inuit language considerations into account, by ensuring the region’s two Inuktut dialects and distinct writing styles were addressed in company communications. Qualified interpreters and modern interpretation equipment were present at Sabina’s meetings, and relevant documents and webpages were translated into both dialects prior to their dissemination in communities. Challenges were presented when certain mining and EA terms did not have equivalent translations in Inuktut. This was mitigated by working with interpreters in advance of meetings to ensure proper understanding, or otherwise being available to discuss these terms with the public in alternative ways. To address Nunavut’s low literacy and educational attainment rates, the engagement program also utilized plain language and varied communication techniques (e.g., visual and audio media), which helped ensure greater uptake of information during the EA process. Traditional Inuit placenames (rather than English names) obtained through TK research were also referenced where available to ensure common understanding by traditional land users.

Likewise, the timing of Sabina’s engagement activities was regularly influenced by community preferences (e.g., avoiding meeting schedules that overlapped with important community events or harvesting/land use activities). In other cases, scheduled meetings often had to be canceled or rescheduled on short notice due to unforeseen community events (e.g., a death in the community or other priorities). Connectivity issues in Nunavut communities also had to be considered when designing Sabina’s community-focused Project website (www.backriverproject.com), by only posting videos and graphics that could be viewed in low internet bandwidth settings and by partitioning large documents into smaller files that were easier to download. The Project website later won an “outstanding achievement” Interactive Media Award, after being judged by members of the Interactive Media Council (www.interactivemediacouncil.org) using criteria pertaining to design, content, feature functionality, usability, and standards compliance, and cross-browser compatibility.

In addition to the above considerations, the logistics of community engagement work in Nunavut presented several challenges to be addressed. For one, there are no roads into Nunavut or between its communities, and nearly all commercial travel is completed by aircraft. These communities are also very remote; for example, the closest permanent community to the Project (Cambridge Bay, pop. 1860) was 300 km away while the furthest that Sabina engaged (Kugaaruk, pop. 1030) was 855 km away. Frequent weather delays and a harsh Arctic climate created additional obstacles to contend with, and the basic infrastructure and services possessed by most communities meant Sabina’s engagement team had to have high preparedness, self-sufficiency, and a continued willingness to adapt. These logistical considerations also ensured the engagement program was higher cost and more time-intensive to implement than similar programs in southern locations.

While challenges to effective engagement exist in Nunavut, careful program design, frequent communication with communities, and attention to local context were key factors that led to successful outcomes for Sabina. Ultimately, many positive comments on the engagement program were received during the EA process. For example:

*…we know that Sabina has been very open and honest in all deliberations and discussions with the Hamlets in the area. Copious meetings have been held in the Kitikmeot region, and the project was fully explained and supported…* (Hamlet of Gjoa Haven 2016).

*…having heard over the couple of years of me being on the Board and ongoing meetings with Sabina and the HTO, I am prepared to support what you’re going after… I want to continue working with you, and let’s keep the community consultation ongoing and also the community involvement* (Cambridge Bay HTO representative *in* NIRB 2016b: 1322–1324).

*…we have got our mining companies [in the Northwest Territories] that are ready to start very near future, but they haven’t done full consultations like what I experienced here… you did your homework thoroughly from the excellent job that you people did…* (Behchoko, Northwest Territories representative *in* NIRB 2017b: 1035).

#### Comprehensive record-keeping and reporting

The importance of record-keeping and reporting to successful engagement outcomes should not be ignored. For one, they demonstrate the extent of consultation performed and provide evidence to support management decisions that are made. They also play a functional role in EA, by validating compliance with various participatory requirements. The Project’s EA engagement record is summarized in Table 2. Records can reveal the number and type of engagements held and/or attempted, who was engaged and on which topics, and how higher priority stakeholders may have been engaged more frequently than others. While Table 2 is obviously a limited snapshot, reporting can be made to include as many details as necessary.

In addition to quantitative consultation records, qualitative tracking of community issues should also be conducted by proponents. Understanding the concerns of communities and opportunities that exist for their resolution is most readily achieved through a multi-faceted engagement program supported by comprehensive record-keeping and analysis. Various databases and qualitative analysis software can be used to help accomplish this.

For example, Sabina’s (2020) community engagement database was developed using QSR NVivo software and contained over 2200 pages of meeting notes recorded during the activities summarized in Table 2. Approximately 165 database topic headings/directories were then created to categorize the stakeholder comments provided in these records. Following a qualitative assessment and comment frequency analysis, a smaller list of 23 priority issues for the EA to focus on was identified. Of these, *caribou* had the highest commenting frequency (335 times) in the most individual meeting records (82), with potential Project impacts noted by a broad segment of community members to be a key issue of concern. The full list of priority issues is presented in Table 3.

**Table 3:** Key issues raised during community engagement for the Project

Theme	Key issues raised during community engagement
Community benefits and engagement	• Inuit culture, harvesting, and livelihoods should not be negatively affected.
	• Kitikmeot communities should receive maximum benefit from the Project.
	• Concern the Project may not be built and/or operate for a long enough period of time. The Project may prematurely shut down, promised benefits will not be realized, and negative socio-economic effects could result.
	• Communities should be regularly engaged throughout the mineral development process.
	• Inuit should play a role in Project-related environmental management and monitoring.
	• Project permitting, regulation and oversight mechanisms are sometimes unclear.
Employment and training	• Preferential employment opportunities should be made available to Inuit from the Kitikmeot Region.
	• Training and apprenticeship programs should be established to help those without mining skills and experience to become meaningfully employed.
	• Mandatory criminal record checks will mean many Kitikmeot residents will not be considered for employment.
	• Youth should be a focus of the employment and training initiatives developed.
	• Routing employees through Yellowknife should be avoided as it leads to issues pertaining to substance abuse, absenteeism, and family instability
	• Programs should be developed to support workers and their families dealing with personal, financial, and employment-related issues.
Environmental management and monitoring	• A comprehensive environmental management and monitoring program should be developed. Key areas of concern include:
	∘ Caribou
	∘ Fish
	∘ Water quality
	∘ Mine tailings and contaminants
	∘ Other wildlife resources
	• Archeological sites within the Project footprint must be protected.
	• Shipping must be conducted safely and responsibly, and impacts on the marine environment must be avoided.
	• Spill training, avoidance, and response capabilities must be developed.
	• Concerns pertaining to the navigability of Bathurst Inlet, placement of dock infrastructure, and the alignment of Project winter roads must be remedied.
	• Cumulative and transboundary effects must be assessed and managed.
	• Guarantees must be in the place that mine closure will be done properly.

It was not enough to simply identify what communities were concerned about; it was also necessary to demonstrate what the company was doing in response. Sabina focused substantial mitigation and management efforts on the issues identified in Table 3 during the EA. These efforts were summarized in relevant volumes of the Final Environmental Impact Statement (FEIS) and FEIS Addendum and in over 30 management plans that accompanied them (Sabina 2015, 2017). Related commitments were then reflected in the Project Certificate issued by NIRB and in the IIBA negotiated with KIA. Sabina also ensured relevant plans and commitments were being communicated back to communities throughout the EA, to demonstrate responsiveness to their concerns and provide additional opportunities for feedback.

To be done well, record-keeping requires dedicated company resources (e.g., time and trained personnel, appropriate software), the use of a consistent and systematic approach, and attention to detail. These records should be as comprehensive as possible and include information on dates, times, locations, participants, topics discussed, decisions reached, and any commitments or follow-up items identified. Proponents should also pay attention to who their intended audience is when developing reports and external communications. While EA reporting is, by necessity, often technical, lengthy, and jargon-ridden, this type of reporting is not overly accessible to Nunavut communities. As noted previously, Sabina made efforts to offer plain language summaries and use other dissemination tools where appropriate.

Without comprehensive record-keeping and reporting, Sabina would have faced significant limitations in its ability to track and respond to community-identified issues, demonstrate the robustness of its engagement program, and substantiate to decision-makers the strong level of community support it developed. This was especially true during the EA’s reconsideration phase. During his final remarks for the Supplementary Final Hearing the NIRB’s Executive Director complemented Sabina in this regard (NIRB 2017b: 1067–1068):

*We realize that the 2016 determination made by the Board was not what the company had wished for, and their efforts to thoroughly address all areas of uncertainty and concern through this additional process have been very much appreciated. The Sabina representatives have done well to respect the Board’s need for transparency, for meaningful public engagement, and for having adequate information to address the questions of the Board, the intervenors, and the public*.

#### Meaningful incorporation of community perspectives and TK

As demonstrated in the previous section, communities in northern Canada often have complex interests and concerns related to mining. These can range from desired economic benefits like employment, training, and business opportunities, to concerns about effects on the natural environment, subsistence harvesting, and community health and well-being. Addressing these issues through meaningful engagement and action is seen as a precursor for sustainable mineral development (see for example papers presented in Southcott et al. 2019). Arctic Council (2019: 17) describes “meaningful” engagement in EA as “a process of participation that is promoting and sustaining a fair and open dialogue. It recognizes the needs, concerns, and values of the public and provides the public with a genuine opportunity to influence decisions made during an EIA.”

In its final decision to approve the Project’s EA, the Government of Canada (2017b: 7) confirmed: “…the other responsible Ministers and I are satisfied that there has been adequate and meaningful consultation with affected Indigenous groups.” Comments received from communities during the EA were also consistent with this perspective. For example:

*… Sabina has consistently engaged with Kitikmeot communities, Federal and Territorial Governments, Hunters and Trappers Associations, the Kitikmeot Inuit Association and other interested groups to gather feedback, provide information and answer question[s]… These consultations have generated significant discussion of the potential opportunities, benefits and challenges associated with developing the [Project]… the overall consensus has been, and continues to be to, to fully support the continued development of this project* (Municipality of Cambridge Bay 2016).

As noted earlier, Sabina’s commitments to addressing key issues raised by stakeholders were described in dedicated sections of the FEIS and FEIS Addendum. Examples of substantive Project modifications made included re-routing the Winter Ice Road to avoid areas of sensitive wildlife habitat, relocating the proposed tailings storage facility off of Inuit-Owned Land, and pursuing a fisheries compensation program based on community-identified priorities. However, Sabina’s use of TK on caribou arguably had one of the greatest influences on Project design.

TK can be defined as “the accumulated body of knowledge, observations, and understandings about the environment, and about the relationship of living beings with one another and with the environment, that is rooted in the traditional way of life of Inuit of the designated area” (NIRB 2018). The importance of TK in northern EA is widely acknowledged (Stevenson 1996; Usher 2000; Diduck et al. 2015; Mulrennan 2015; Sinclair and Doelle 2015; Barry et al. 2016; Mitchell 2019). This is because Indigenous peoples are known to possess detailed knowledge of their local environments and strategies for managing those environments due to years of intensive land use and observation. TK is especially useful in locations like the Canadian Arctic where scientific information on certain species and environmental topics may be lacking. TK can thus be used to complement existing sources of scientific information, while also acting as a distinct and new source of information when other sources are limited.

Caribou TK studies provided Sabina with information on historical and contemporary baseline conditions (e.g., pertaining to caribou ecology, health, migration, calving, and Inuit harvesting practices), and revealed potential future overlaps between the Project and changing caribou migration and calving areas. Inuit also identified reactions caribou would likely exhibit to mining-related disturbance, and offered mitigation suggestions to Sabina.

This information informed the development of several Project mitigation measures. This included an active year-round caribou monitoring program, creation of operational setbacks and safety zones protective of caribou, and rapid and planned operational shutdown procedures to be enacted if caribou calving/post-calving shifts occur. Sabina also committed to establishing both an Inuit Environmental Advisory Committee (IEAC) and Caribou Technical Advisory Group to provide additional oversight of the Project.^6^ Together these resulted in what the Government of Canada (2017b: 7) later called “…some of the most stringent caribou protection measures ever developed for a mine development in the Arctic”. A member of the Kugluktuk HTO likewise noted:

*Your program is a lot more in-depth than it was at the original hearings. I think you guys have gone way above and beyond what is being called of you… We still support Sabina, and we still will. We know your mitigation measures out there are way above and beyond what is being called of you guys* (Sabina 2020 – December 2016 meeting with the Kugluktuk HTO).

Sabina followed established best practice guidance on the collection and use of TK during the EA (e.g., ITK and NRI 2007; Tobias 2009; Armitage and Kilburn 2015). This included use of informed consent and rigorous data collection protocols in addition to data verification activities. This also involved collaborating with community organizations in designing and executing TK studies for the Project. For example, TK partnerships with both the KIA and Kugluktuk HTO were established through signed agreements and/or licensing arrangements. These partnerships guaranteed Inuit oversight of the TK research process and ownership of TK that was collected, while also securing access to key information for Sabina during the EA. The majority of TK was made available through Sabina’s TK licensing arrangement with the KIA. In this case, the KIA was responsible for identifying the Project’s TK data needs, planning the research program, identifying research participants and conducting data collection/verification activities, and preparing final reports. KIA also retained ownership of the data and acts as a steward for its long-term storage and use.

Likewise, diverse sources of TK were accessed to ensure community perspectives were adequately being captured and existing data gaps were being addressed. These included interviews, workshops, and mapping exercises; existing databases; published TK in the literature; and other sources (e.g., site visits, land use workshops, government harvest study results). Sabina’s TK studies were also widely scoped, with numerous wildlife and Inuit land use topics being investigated in addition to caribou. Praise for Sabina’s TK program was offered by two NIRB board members during the Supplementary Final Hearing:

*I would like to compliment Sabina as well in their efforts to include traditional knowledge and Inuit Qaujimajatuqanjit, and that is a first for any company to include those two* (NIRB board member *in* NIRB 2017b: 135).

*… I think because you are dealing with a community-based organization, you have a better understanding of how their lifestyles and how they interpret environment, wildlife, and all aspects of their lives around them… I just wanted to again acknowledge that you guys have come very close to bridging that big, large gap between traditional knowledge from a scientific point of view…* (NIRB board member *in* NIRB 2017b: 127–128, 133).

To be clear, achieving this outcome was neither easy nor quick. Relationships with key stakeholders took time to develop, and TK study priorities and expectations needed to be clearly defined and agreed upon (supported by formal agreements in some cases). Substantial time, personnel, and resources were also required to properly execute the TK program and develop relevant mitigation. Sabina’s approach to TK also introduced a measure of corporate risk, as control over the TK research process and its outputs no longer resided solely with the company, but was shared with its community partners instead. These obstacles and risks were heavily outweighed, however, by the potential for Project delays caused by not meaningfully incorporating TK into the EA process.

#### Long-term relationships, partnerships, and local benefits

Some proponent-led engagement programs have been criticized for focusing only on short-term objectives (e.g., an EA approval). However, communities often remain concerned about future impacts, desire lasting socio-economic benefits, and expect proponents to be available to address their issues throughout the development process. When communities are doubtful these matters will be addressed, their likelihood of supporting a project will wane.

With this in mind, Sabina took a long-term approach to working with surrounding communities. Foremost, the company ensured mechanisms were in place for communities to engage with Sabina both during and following the EA. This included the development of a public-facing Community Involvement Plan to describe Sabina’s ongoing consultation commitments, establishing an IEAC to share information and receive feedback from Inuit as operations advanced, and initiating an Implementation Committee with KIA to oversee the functioning of the IIBA.

Project monitoring requirements in Nunavut are also extensive and include regular, post-EA, opportunities for stakeholders to provide input on the Project. This builds additional trust in the EA process, by providing ongoing oversight and accountability for commitments made by proponents. Linked to compliance with a NIRB-issued Project Certificate and other approvals, proponents are required to submit detailed annual monitoring reports to NIRB on environmental and socio-economic aspects of their operations. NIRB then disseminates these reports to stakeholders for comment (which proponents are expected to address), conducts annual community engagement on its monitoring activities, and issues final recommendations to proponents where necessary (NIRB 2013). Several community-focused terms and conditions are included in Sabina’s Project Certificate, including those ensuring TK and local community knowledge are integrated throughout the monitoring program, that socio-economic conditions and impacts in surrounding communities are monitored annually, and that various multi-stakeholder forums are established to monitor important Project trends and outcomes.

A series of long-term community partnerships were also developed by Sabina in support of Project advancement. In addition to the TK partnerships highlighted previously, additional partnerships were established with the KIA to deliver community benefits and with the Kugluktuk HTO to execute the Bernard Harbour Restoration Project. Termed “transformational engagement” by Bowen et al. (2010), partnerships typically involve joint project management and decision-making, frequent two-way communication and dialogue, and the sharing of benefits and outcomes with local communities. Partnerships require a substantial commitment from all participants, but can meaningfully contribute to a project’s success when utilized properly.

For example, during the EA the Kitikmeot Region communities expressed a strong desire for socio-economic benefits and Sabina spent considerable effort ensuring appropriate opportunities would be created. Benefits commitments were initially captured in various socio-economic management plans found in the FEIS, then formalized through an IIBA with the KIA. IBAs are privately negotiated, legally enforceable agreements that establish formal relationships between Indigenous communities and industry proponents. They are intended to address potential adverse effects of development activities on Indigenous communities by providing some compensation for these activities and to ensure these communities acquire benefits from development activities occurring on their traditional territories (Kielland 2015). Noteworthy provisions of the Project’s IIBA and associated land tenure agreements include (Sabina and KIA 2018):

- 1% net smelter return royalty and 6.7 million Sabina shares to the KIA.
- Inuit employment, training and education, and business opportunity commitments.
- A Regional Wealth Creation initiative, to create new long-term jobs outside of mining that expands and diversifies the Kitikmeot economy (supported by an initial Sabina investment of $4 million).
- Annual payment to the KIA of up to $1 million to cover costs of implementing the IIBA and land tenure agreements.
- Water and wildlife compensation agreements including additional payments if Sabina fails to implement caribou mitigation commitments made to NIRB.
- Land use licenses, advanced exploration leases, and commercial leases for the Project.
- KIA consent to operations and confirmation they were adequately consulted on the Project.

KIA’s President noted “these agreements allow a mine to be built and operated on Inuit Owned Land and will provide significant social and economic opportunities and benefits to Inuit of the Kitikmeot Region. Sabina has been very professional in these negotiations. We wish them success in their development plans for the mutual benefit of Sabina shareholders and Kitikmeot Inuit” (Sabina and KIA 2018). A sustainable approach to mining increasingly demands that meaningful benefits be provided to nearby communities; without agreed-upon, long-term benefits community support is often not provided (Veiga et al. 2001; Prno 2013; Fitzpatrick and McAllister 2015; Mulrennan 2015; Southcott et al. 2019). The Project’s IIBA and associated land tenure agreements thus created not only a long-term partnership with KIA that allowed the Project to proceed, but serve as key mechanisms through which lasting community benefits will be delivered in the region.^7^

The Bernard Harbour Restoration Project was another community partnership undertaken by Sabina. Bernard Harbour is a traditional Inuit fishing area located outside the Hamlet of Kugluktuk. Once a place of plenty, due to changing climate conditions there has been a profound decrease in the number of Arctic Char traveling from the ocean to spawn upstream due to obstructions in their path. Looking for ways to help recover fish stock at Bernard Harbour, the Kugluktuk HTO came up with a plan to restore the nearby creek and revitalize the number of Char present. Collaboration was key to this project. Blending TK with modern resources, the parties worked together on scientific baseline studies, TK research, and restoration activities, and ultimately saw the number of fish increase in the area. Details on the relationship between the two parties in executing the project were first agreed upon through consultation and then finalized in a formal agreement.

This partnership has served mutual aims. Firstly, the desires of Kugluktuk community members and the HTO to restore a traditional fishery are being addressed, and training has been provided that supports local management of this resource into the future. Secondly, restoration activities helped satisfy Fisheries Act offsetting requirements for the Project. Due to proposed lake dewatering and stream course alteration activities at Back River, Canadian law required Sabina to compensate for, or “offset”, all fisheries-related impacts. Unprecedented as fisheries mitigation in Canada at the time, the Bernard Harbour Restoration Project subsequently became an important component of the Project’s EA and fisheries-related authorizations. The Kugluktuk HTO commented on the success of the project during the Supplementary Final Hearing:

*In regards to Bernard Harbour, I do commend Sabina for the continual work with the HTO… The Bernard Harbour Fish Restoration Project, along with other government agencies, the HTO, a mining industry just shows how an Inuit organization such as our HTO can work hand in hand with western science, industry, and traditional knowledge* (Kugluktuk HTO Chairperson *in* NIRB 2017b: 669 and 978).

Substantial investments of time, personnel, and capital were required to execute Sabina’s partnerships. This suggests a need for proponents to be selective and strategic when developing these long-term initiatives, in order to focus efforts and ensure the appropriate use of limited corporate resources. Furthermore, not every local organization has the same level of influence, capacity, or desire to participate in a partnership, and some stakeholders are better suited to alternative forms of engagement. The KIA and Kugluktuk HTO, for instance, already had established governance roles in Nunavut, possessed the capacity to deliver on issues within their mandates, and were widely recognized as key representatives of their communities. There should also be a reasonable expectation of mutual aims being achieved prior to fully committing a company’s resources to a partnership. This is especially so where important project outcomes will be dependent on successful partnership execution, as they were for Sabina. Partnerships can also require a measure of uncertainty to be embraced, especially when novel programs are being developed and when decision-making authority is being shared with outside partners. Partnerships are thus one component of a larger engagement program (see Table 1).

Likewise, community socio-economic development objectives and capacity are necessary to understand when implementing benefits programs. Many local development initiatives have failed when community needs and realities were not properly considered (Agrawal and Gibson 1999; Cleaver 1999; Mehta et al. 1999). There is also no standard formula for what these programs should entail. Feedback from communities and experienced practitioners, existing IBAs (where public), and published guidance (e.g., Gibson and O’Faircheallaigh 2015; Bradshaw et al. 2019) may be helpful planning tools in this regard. Enforcement and dispute resolution mechanisms are also useful to address and can lead to enhanced stakeholder trust in the commitments being made.

## Concluding Remarks

Community engagement serves many aims. For mining proponents in the Canadian Arctic, it functions as a key platform to address local concerns, build community support, and help secure project approvals. As demonstrated throughout this paper, broad community support was achieved by Sabina through the course of its engagement program for the Back River Project. An EA approval for the Project was also issued after an exhaustive review process that placed substantial focus on community participation and local knowledge integration.

Effective community engagement was accomplished by Sabina, in large part, through engaging early and often using a context-specific approach; comprehensive record-keeping and reporting; the meaningful incorporation of community perspectives and TK; and focusing on long-term relationships, partnerships, and local benefits. Similar insights have been reflected elsewhere in the best practice literature and Sabina’s success lends further credibility to their application in Canada’s Arctic. However, best practices are often presented in a general and broadly applicable manner. Sabina’s experiences strongly suggest the need for proponents to adapt and tailor their community engagement programs according to their individual circumstances.

Company-specific factors must also be considered. The authors found having the strong support of senior personnel (i.e., at the Board of Directors and executive levels) was vital to ensuring appropriate program resources were made available and community perspectives were being considered throughout corporate decision-making. Likewise, the skills and qualifications of the engagement team members themselves were important; northern community experience, inter-personal and inter-cultural capabilities, familiarity with best practices, and a capacity to execute responsibilities in challenging circumstances were some of the traits that contributed to successful outcomes. Having consistent personnel represent the company was also important and encouraged local familiarity, relationship continuity, and enhanced opportunities for dialogue and trust-building with communities.

Elements outside a company’s influence can also affect outcomes. Sabina’s success was facilitated by the jurisdiction in which it operated (Nunavut), including its settled land claim and robust regulatory regime. This created enhanced certainty for parties involved in the EA process by establishing comprehensive participatory requirements and oversight mechanisms, and by clearly delineating where Inuit had authority over Nunavut’s lands and resources. Without strong institutional arrangements such as these (and the organizations to uphold them), communities would have much less influence in Nunavut’s EA process and weaker incentives would exist to address their concerns.

In addition, communities of the western Kitikmeot Region had previous experience with mining. This meant residents were knowledgeable about resource development prior to Sabina’s arrival and were better prepared to engage Sabina on complex issues early in the EA process. Previous exposure to the opportunities offered by mining, in addition to persistent regional socio-economic challenges such as high unemployment, may have also created a more supportive context from early on. The promise of jobs and other economic opportunities was often cited by communities as a key reason for supporting the Project during the EA (e.g., NIRB 2016b and 2017b; Sabina 2020).

However, not all communities can be expected to have similar views on mineral development, and some projects may never be able to reconcile the concerns voiced by residents. While this reality must be acknowledged, insurmountable “project-stopper” disputes are not overly common in the Canadian Arctic and many examples of compromise and accommodation exist. To be clear, Sabina did not receive the support of 100% of its stakeholders, and no mine likely ever will. The goal of mineral developers should thus be to meaningfully engage all key parties, substantially address relevant concerns where practical, and build community support when possible. Ultimately, government and regulatory officials need to balance any competing considerations when making their final decisions.

Challenges and setbacks are also bound to arise in any engagement program. Even with the substantial community support Sabina had in Nunavut leading up to the initial Final Hearing, for example, a negative recommendation was still issued by NIRB over concerns about potential significant adverse effects. Transboundary issues related to caribou were a major focus for NIRB and the views of Northwest Territories groups opposing the Project are believed to have played an important role in the Board’s initial decision. Leading up to this, Sabina had placed substantial focus on engaging Inuit and prioritizing Nunavut-based issues during the EA. This approach was purposeful and justifiable on several grounds, but likely also resulted in some Northwest Territories residents feeling insufficiently engaged by Sabina. While this suggests more fulsome engagement with Northwest Territories groups could have been conducted to help avoid these issues, other factors should also be considered.

For one, it must be kept in mind this was a Nunavut-based project with mineral resources located on Inuit Owned Land established through a settled land claim. The Project thus fell under the jurisdiction of the NA and the EA process outlined in it. In 2012, the federal government also confirmed the Project was to be assessed through a NIRB-led Part 5 review under the NA and not a Part 6 review, which would have required the establishment of a federal EA panel.^8^ Furthermore, Project effects were expected to be mostly concentrated in Nunavut and on Inuit communities. These combined factors necessitated the strong prioritization of Inuit issues in the EA. Secondly, the Government of Canada (2017a) uncovered various deficiencies in the initial conclusions reached by NIRB before returning the assessment to the Board for further review.^9^ We acknowledge different stakeholder views were expressed on this matter; some individuals saw this as a reasonable rebuke of NIRB’s handling of the file, while some saw it as federal interference in the work of a land claims-enshrined co-management board. We view this in an alternative light and consider the case a successful application of the NA and a recognition of Inuit rights. This is because the priorities of regional Inuit were ultimately satisfied through the process. Moreover, the NA’s signatories explicitly envisioned the need for an executive arbiter of issues when they included provisions for federal ministerial oversight of Nunavut’s EA process (see Article 12.5.7 of the NA).

While Sabina addressed NIRB’s concerns through additional mitigation and consultation, the Project’s enduring community and stakeholder support in Nunavut arguably played an important role in overturning the initial recommendation. A continued commitment to meaningful engagement and willingness to adapt ultimately assisted Sabina in advancing the Project through the EA phase.

There is now a growing body of successful case study evidence and best practice guidance available for developers to consider when implementing engagement programs. This paper adds to this body of knowledge, by discussing the successes and challenges encountered by one mineral developer implementing a community engagement program in the Canadian Arctic. It is hoped these insights will contribute to more effective engagement programs in the mining industry, enhanced consideration of stakeholder concerns in EA, and the improved well-being of nearby communities.

## Footnotes

1 While these Nunavut communities were the focus of Sabina’s activities, relevant transboundary Indigenous groups in the Northwest Territories were also engaged due to overlapping land use and harvesting interests (primarily related to caribou).

2 These benefits include ownership of nearly 18% of the land in Nunavut (termed “Inuit Owned Land”), including mineral rights to 2% of these lands; a cash settlement of $1.2 billion; and the creation of the Territory of Nunavut in April 1999 with a government dedicated to serving the interests of all Nunavummiut.

3 For example, public consultation is listed as the first of 11 “minimum requirements” for proponents preparing an environmental impact statement (EIS) in Nunavut. NIRB requires mining proponents to consult meaningfully with all potentially affected communities and link community input that is received with tangible effects on project proposals. NIRB also requires Traditional Knowledge (TK) (the terms “Inuit Qaujimaningit” and “Inuit Qaujimajatuqangit” are also used in Nunavut) to be incorporated substantially throughout an assessment, and the Board’s work continues to be guided by Inuit societal values. Detailed consultation requirements for proponents are provided in NIRB (2020) and within project-specific EIS guidelines.

4 These are available on NIRB’s public registry (https://www.nirb.ca/project/124149), as are letters from the KIA, Government of Nunavut, and Government of the Northwest Territories that supported Project advancement or requested the Minister return the Project to NIRB for further review. In particular, KIA’s August 24, 2016 letter noted “Inuit support for the Project is firm across the Kitikmeot Region…”.

5 While several meetings had been completed prior, Sabina’s community engagement program formally commenced in 2012 and coincided with a Project Description being submitted to the Nunavut Planning Commission and NIRB. Submission of a Project Description commences the Nunavut EA process for mining projects. Additional stages in the EA process typically include submission of draft and final EISs, concordance and technical reviews, technical meetings, community meetings and roundtables, and final hearings. Opportunities for stakeholder input are provided throughout this process, which proponents are expected to address through additional submissions (NIRB 2020).

6 As reflected in Sabina’s Wildlife Mitigation and Monitoring Plan (WMMP).

7 Article 26 of the NA necessitates the negotiation of an IIBA for major development projects (e.g., mining projects) located on Inuit Owned Land before operations begin. Under the NA, various land-use leases and permits were also required from the KIA before the Project could proceed.

8 In making the decision for a Part 6 review two factors are considered: (1) whether the project proposal involves matters of important national interest, and (2) whether the project proposal is to be carried out partly within and partly outside the Nunavut Settlement Area (Barry et al. 2016).

9 For example, the Government of Canada (2017a: 3) noted “…it is premature to conclude that the Project would lead to unacceptable or unmanageable ecosystemic or socio-economic impacts. There are a number of areas where there was insufficient information presented in the report to support the conclusions of the Board, and where further information is required so that the ministers may understand the rationale behind the conclusions presented by the Board prior to making a decision on whether the project should proceed. This is particularly important in areas where the Board has expressed a lack of confidence or significant uncertainty while the report and the broader record indicates that many of the participants (including Indigenous and non-Indigenous witnesses and subject matter experts) expressed confidence that the measures presented could mitigate and manage potential adverse effects to an acceptable level. A deeper inquiry into the issues identified by the Board might point to sound management approaches.”
